# Physician-brief advice for promoting smoking cessation among cancer patients on treatment in low and middle-income countries: a scoping review

**DOI:** 10.1186/s12885-024-11872-z

**Published:** 2024-01-30

**Authors:** Olayinka Stephen Ilesanmi, Babalola Faseru, Aanuoluwapo Adeyimika Afolabi, Olukemi Odukoya, Olalekan Ayo-Yusuf, Folahanmi Akinsolu, Akindele Olupelumi Adebiyi, William K. Evans

**Affiliations:** 1 Africa Centres for Disease Control and Prevention, Addis Ababa, Ethiopia; 2 Department of Population Health, University of Kansas Medical Center, Kansas City, US; 3 Department of Family Medicine and Community Health, University of Kansas Medical Center, Kansas City, US; 4 Department of Medicine, University of Ibadan, Ibadan, Oyo State Nigeria; 5 Technical and Strategic Research Directorate, MSI Nigeria Reproductive Choices, Abuja, Nigeria; 6 Department of Community Health and Primary Care, College of Medicine, University of Lagos, Lagos, Nigeria; 7 School of Health Systems and Public Health, University of Pretoria, Pretoria, South Africa; 8 Department of Public Health, Lead City University, Ibadan, Nigeria; 9 Department of Community Medicine, University of Ibadan, Ibadan, Oyo State Nigeria; 10 Department of Oncology, McMaster University, Hamilton, ON Canada

**Keywords:** Physicians, Physician-brief advice, Smoking, Smoking cessation, Low and middle-income countries

## Abstract

**Introduction:**

Physician-brief advice has been utilized in high-income countries to promote smoking cessation among cancer patients. Empirical evidence on its effectiveness among cancer patients in low and middle-income countries (LMICs) is lacking. The gap could be due to inadequate training, and competing healthcare priorities, leading to insufficient implementation of targeted smoking cessation interventions in oncology settings. We undertook this scoping review to determine if physician-brief advice is effective in promoting smoking cessation among cancer patients in LMICs.

**Methods:**

We conducted a literature search of all relevant articles across five databases: Cochrane Central Register of Controlled Trials, Cochrane Library (Tobacco Addiction Group trials), World Conference on Lung Cancer proceedings, PubMed, and Google Scholar up to November 2023, using pre-defined inclusion criteria and keywords. The study population was cancer survivors in LMICs, the intervention was smoking cessation advice by a physician in a clinic or oncology center during a consultation, and the outcome was the effect of smoking cessation programs in discontinuing smoking among cancer survivors in LMICs.

**Results:**

Overall, out of every 10 cancer patients in LMICs, about seven were smokers, and one-half had received physician-brief advice for smoking cessation. Physician-brief advice was more likely to be delivered to patients with smoking-related cancer (Cohen’s d = 0.396). This means that there is a noticeable difference between patients with smoking-related cancer compared to those with cancer unrelated to smoking. Smoking cessation failure was due to the inability to cope with the symptoms of withdrawal, missed smoking cessation clinic visits, mental health disorders, limited time and resources, and minimal patient-physician contact.

**Conclusion:**

There is very little literature on the frequency of use or the efficacy of physician-brief advice on smoking cessation in LMICs. The literature suggests that cancer patients in LMICs have low self-efficacy to quit smoking, and smoking cessation is rarely part of cancer care in LMICs. Physicians in LMICs should be trained to use motivational messages and good counseling techniques to improve smoking cessation among cancer patients. Policymakers should allocate the resources to implement physician-brief advice and design training programs for physicians focusing on physician-brief advice tailored to cancer patients.

## Introduction

Can the link between smoking and cancer be fully understood without considering the effectiveness of physician-led interventions? Absolutely not! In the realm of healthcare, the link between smoking and cancer stands as a stark testament to the intricate interplay between personal choice and the formidable specter of disease. Amidst this complexity, the role of physicians transcends the realms of diagnosis and treatment, expanding into the domain of profound influence and transformative guidance [1]. For cancer patients, the connection between smoking and their condition is not merely theoretical but deeply personal, entwined with their journey through illness and survival. It is within this fragile juncture that the power of physician-brief advice on smoking cessation assumes profound significance [2]. Physicians, as guardians of health and bearers of knowledge, possess a unique opportunity to shape the narratives of their patients’ lives [3]. A simple yet poignant piece of advice, delivered with empathy and understanding, can serve as the catalyst for a profound transformation. It is a moment where medical expertise converges with compassionate mentorship, urging individuals to redefine their relationship with tobacco and, in doing so, potentially altering the trajectory of their illness. In this intricate dance between medical wisdom and personal choice, the impact of physician-brief advice not only echoes within the chambers of individual lives but reverberates through the corridors of public health, underscoring the pivotal role of healthcare providers in the battle against both cancer and the enduring habit of smoking. This is very important given the increasing occurrence of cancer in many countries.

The incidence of cancer is increasing across low and middle-income countries (LMICs) [3]. By 2030, it is projected that 75% of the world’s cancer deaths will occur in LMICs [4]. Physician-brief advice on smoking for cancer patients is an intervention based on research that aims to increase smokers’ attempts to stop smoking [1]. The three steps of physician-brief advice are to “Ask” patients about their tobacco use, “Advise” them that the best method of quitting is with a combination of medication and behavioral support, and “Act” by assisting them to make a quit attempt using available cessation supports [1]. Physician-brief advice was developed by the National Centre for Smoking Cessation Training (NCSCT) in the United Kingdom [1]. Despite the standardization of the physician brief advice as a useful tool to quit smoking and though many cancer patients make attempts to quit smoking after being diagnosed with cancer, more than 50% of cancer survivors continue to smoke [5, 6]. Thus, patients on cancer treatment require physician-tailored smoking cessation interventions. It has been reported that smoking cessation interventions can reduce smoking by a maximum of 50% at 6 and 12 months of follow-up compared to the baseline period [5]. While some studies have reported the effectiveness of physician-brief advice in promoting smoking cessation, other studies have identified that one-fourth of cancer patients who received physician-brief advice lacked interest to quit smoking [7, 8]. LMICs face unique challenges and characteristics that are particularly relevant to smoking cessation and the implementation of physician-brief advice programs. Some of these challenges include low health literacy about the risks of smoking and the benefits of cessation, high burden of other health issues, and the limited availability of healthcare resources [9].

While physician-brief advice is a universal component of cancer care in high-income countries (HIC) [9, 10], robust information on the effectiveness of physician-brief advice in LMICs remains uncertain. We, therefore, undertook this scoping review of the literature to fill this research gap to understand the effectiveness of physician-brief advice in promoting smoking cessation among cancer patients in LMICs. Scoping reviews are used to map key concepts, types of evidence, and research gaps in a particular field. A scoping review is an appropriate methodology for this study because it provides an overview of the existing literature and identifies gaps in knowledge.

## Methods

A scoping review was conducted to appraise and summarize all available empirical evidence on the effectiveness of physician-brief advice in promoting smoking cessation among patients receiving oncology care in LMICs. The overall search period lasted for three months at two different instances. The review was undertaken to determine if physician-brief advice helped to reduce tobacco smoking among cancer patients receiving oncology treatment in LMICs.

There was an exploratory analysis before making the decision on which databases to use in the review. As a result, we searched the Cochrane Central Register of Controlled Trials, Cochrane Library (Tobacco Addiction Group trials), World Conference on Lung Cancer proceedings, PubMed, and Google Scholar up to November 2023. The literature search included articles in English language only and was conducted between June-December 2022, and repeated between October-November, 2023 to identify new publications (if any). We did not have restrictions at all on the year of publication, patients’ age, and length of follow-up. In addition to the original literature search, the reference list of retrieved articles was examined to identify eligible articles fit for inclusion in this review. Two authors (OSI and AAA) conducted the literature retrieval. All retrieved articles were screened manually, and duplicates were removed. OSI and AAA proceeded to independently screen the titles, abstracts, and full texts of retrieved articles for inclusion in the review. To resolve disagreements in the inclusion of an article, the authors referred to the inclusion and exclusion criteria. Where disagreements remained unresolved, BF mediated and made the final decision.

### Research question

Is physician-brief advice effective in promoting smoking cessation among cancer patients in LMICs?

### PICO element

#### Population

Participants were cancer survivors in LMICs. Only survivors that had received smoking cessation advice from a physician were included in the study.

#### Intervention

- Who: The intervention must have been provided by physicians which could be general practitioners, oncologists, or practitioners in other subspecialties of medicine.
- Where- A healthcare facility; an outpatient clinic setting or oncology center.
- What:
  i. The type of intervention provided: Physician-brief advice.
  ii. Study design: All study designs were included: RCTs, cross-sectional studies, case-control studies, cohort studies, and qualitative studies.

d. How- Patient-physician interaction, or physician-led communication during a consultation session.

#### Outcome

The effect of smoking cessation programs in discontinuing smoking among cancer survivors in LMICs was the main outcome of the review.

### Inclusion criteria

The inclusion criteria for articles were as follows:

- Provision of oncology care.
- Delivery of counseling or advice by physicians.

### Exclusion criteria

- All studies conducted among cancer patients or survivors in high-income countries.
- Absence of a definite follow-up period for the physician-brief advice intervention.
- Articles containing protocol on physician advice for smoking cessation among cancer patients.

### Boolean operators used in the literature search

AND

OR

## Medical subject headings (MeSH) terms used in the literature search

Smoking cessation.

Neoplasms.

Prophylaxis.

Survivors.

Counseling.

Table 1 describes the keywords and search strings used in the literature search.

**Table 1:** Keywords and search strings used in the literature search

Keywords	Search string
Smoking cessation, Tobacco cessation, Quit smoking, Cancer patients, Oncology patients, Cancer survivors, Physician intervention, Physician advice, Brief advice, Healthcare provider support, Smoking cessation programs, Nicotine replacement therapy, Behavioral interventions, Support groups, Counseling, Tobacco dependence treatment, Health promotion, Cancer treatment outcomes, Health behavior change	((((“smoking cessation“[MeSH Terms] OR (“smoking“[All Fields] AND “cessation“[All Fields]) OR “smoking cessation“[All Fields]) AND “mh or“[All Fields]) AND “stopping smoking“[Title/Abstract]) OR “giving up smoking“[Title/Abstract] OR “quitting smoking“[Title/Abstract] OR “prevention*“[Title/Abstract] OR “smoking cessation intervention“[Title/Abstract] OR “single therapy“[Title/Abstract] OR “Brief Physician Advice“[Title/Abstract] OR “Behavioral counseling“[Title/Abstract] OR “Telephone counseling“[Title/Abstract] OR “Self-help materials“[Title/Abstract] OR “combined modality therapy“[MeSH Terms] OR “Brief advice“[Title/Abstract] OR “education*“[Title/Abstract] OR “counseling*“[Title/Abstract] OR “guidance*“[Title/Abstract] OR “Recommendation“[Title/Abstract] OR “instruction*“[Title/Abstract] OR “assistance*“[Title/Abstract] OR “information*“[Title/Abstract] OR “suggestion*“[Title/Abstract]) AND (“cancer treatment“[Title/Abstract] OR “Neoplasm treatment“[Title/Abstract] OR “Cancer care“[Title/Abstract] OR “cancer therapy“[Title/Abstract] OR “radiotherapy*“[Title/Abstract] OR “chemotherapy*“[Title/Abstract] OR “chemoprevention*“[MeSH Terms] OR “chemoprophylaxis*“[Title/Abstract] OR “prophylaxis*“[Title/Abstract] OR “prevention*“[Title/Abstract]) AND (“Low income“[Title/Abstract] OR “low-middle income“[Title/Abstract] OR “upper-middle income“[Title/Abstract] OR “middle income“[Title/Abstract] OR “countries*“[All Fields] OR “Africa*“[All Fields]) OR “group“[Title/Abstract])

**Fig. 1:**
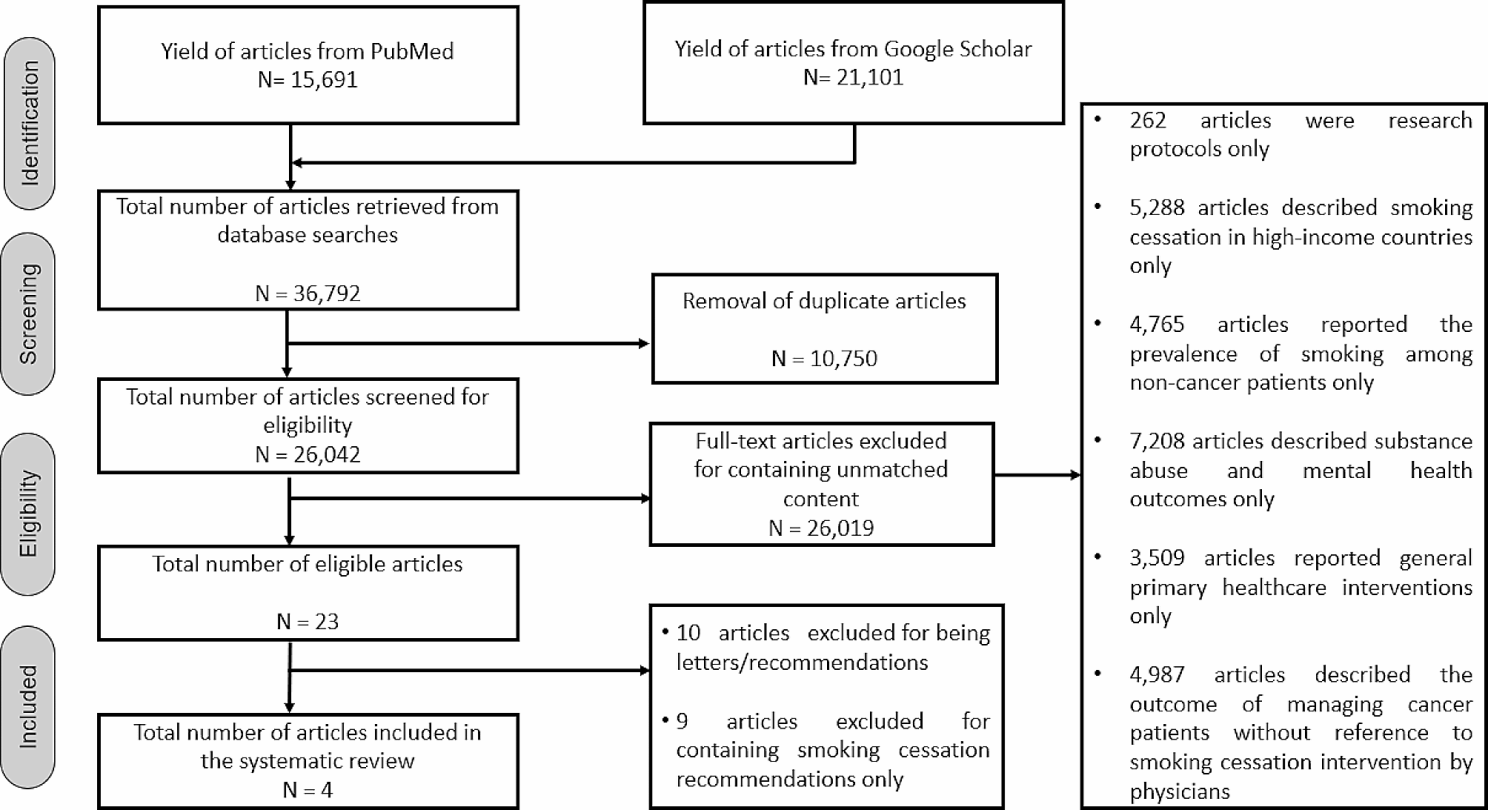
Flowchart describing the literature retrieval strategy for the scoping review

Figure 1 shows the flowchart describing the literature search process. Overall, 37,854 articles were retrieved. Following the removal of 10,750 duplicate articles, 27,104 articles were screened for eligibility. Of this total, 27,090 articles were excluded due to unmatched content, thus streamlining the total number of eligible articles to 27. This was followed by the removal of 23 articles due to one of the following reasons:

i. The articles were protocols of studies that have not been conducted.
ii. The studies were conducted among mixed populations, e.g., African Americans, and they did not report the physicians’ role in promoting smoking cessation among cancer patients.

Overall, four articles were included, and the period of publication spanned twelve years: between 2011 and 2022. Three studies were conducted in Jordan, and one was conducted in South India.

## Results

Table 2 summarizes the literature included in the scoping review. To estimate abstinence rates and describe reasons for failure to quit smoking among patients visiting a smoking cessation clinic in a comprehensive cancer centre in Jordan, Hawari and colleagues conducted a combined retrospective and prospective study among 156 cancer patients referred to receive counseling and pharmacological treatment at the smoking cessation clinic. The overall smoking cessation rate after the 12-month review period was 21.2%, and the smoking cessation rate was highest among those who smoked ≤ 10 cigarettes daily [11].

**Table 2:** Summary of literature included in the scoping review

S/N	Author	Objective	Study population (Location)	Methods	Category of physician (if stated)	Smoking cessation rate	Reasons for failing to quit smoking	Implications
i.	Hawari et al., 2012 [11]	To estimate abstinence rates and describe reasons for failure to quit smoking among patients visiting a smoking cessation clinic in a comprehensive cancer centre	156 cancer patients referred to receive counseling and pharmacological treatment at a smoking cessation clinic (Jordan)	**Study design**: i. Retrospective medical chart review of patients referred to the smoking cessation clinic between February 2008 and October 2009 ii. Prospective patient follow-up via phone calls	Oncology physicians	Overall smoking cessation rate: 21.2% The likelihood of quitting smoking reduced with increasing smoking intensity at 12-month follow-up: None of the heavy smokers that smoked 21 cigarettes or more daily was able to quit; 2% of medium-intensity smokers (those who smoked 11–20 cigarettes daily) had successfully quit smoking; and 44.4% of those who smoked the least (≤ 10 cigarettes daily) were abstinent at the 12-month follow-up.	i. Personal or professional stressful situations ii. Inability to handle withdrawal. iii. The belief that quitting smoking has no value. iv. Depression v. Lack of support vi. Adverse side effects of medication vii. Weight gain	Interventions to improve smoking cessation rates among cancer patients should be more focused on helping patients handle cessation-related stress, and on the review/ adjustment of the medication regimen to improve withdrawal symptoms
ii.	Hawari et al., 2013 [12]	To evaluate smoking cessation in a challenging group of cancer patients (medium-to-heavy smokers) visiting an out-patient smoking cessation clinic (SCC) in a cancer center	201 patients smoking > 9 cigarettes per day (CPD) and referred to the SCC between June 2009 and May 2012 (Jordan)	**Study design**: Longitudinal (3-, 6- and 12- month) follow-up by phone/clinic visit **Tool/Data source**: Medical records	General practitioners		i. Prevalence of smoking abstinence at 3 months: 23.4% ii. Prevalence of smoking abstinence at 1 year: 6% iii. Reasons cited for smoking cessation failure included: inability to handle withdrawal (32.4%), no value seen in quitting (19.6%), and depression (14.7%)	i. Tobacco-related damage has already been done and quitting has no value. These results are relevant to note when considering the content of counseling sessions and how it should evolve progressively as the nature of challenges faced by the smoker change ii. Reasons for failure to abstain should be used to tailor counseling practices
iii.	Matthew et al., 2019 [13]	To investigate the status of quitting and examining the motivations for tobacco cessation after receiving a head and neck cancer diagnosis	71 cancer patients who were tobacco users aged ≥ 25 years (India)	**Study design**: Cross-sectional study design **Tool**: Distress Thermometer	Oncology physicians		67.6% of cancer patients who had been smoking prior to cancer diagnosis quit smoking due to oncologists’ advice	Strong and frequent advice from oncologists to cancer patients on the need to stop smoking is an important psychosocial factor in promoting smoking cessation among cancer patients
iv.	Hawari et al., 2019 [14]	To evaluate receipt of care at a smoking cessation clinic and the effect of assisted abstinence through the smoking cessation clinic on short-term (two-year) survival after a cancer diagnosis	3,403 cancer patients diagnosed between 2009 and 2016, who also were cigarette smokers, and who received full treatment at King Hussein Cancer Center (Jordan)	**Study design**: Retrospective cohort study design **Tool/Data source**: Cancer registry and smoking cessation clinic data	General practitioners	There was a higher survival advantage for those who reported abstinence at only one follow-up point at thee smoking cessation clinic which may indicate a harm reduction effect.	i. Prevalence of cancer patients seen at the smoking cessation clinic: 21% ii. Cancer patients that had never been seen at the smoking cessation clinic or seen after a year from diagnosis had nearly 3 times higher hazard rates compared to those seen more than once at the smoking cessation clinic whose records indicated abstinence on at least two of their 3-, 6- or 12-month follow-up visits) [Hazard ratio: 2.8; 95%CI: 1.7–4.6] iii. Cancer patients who abstained from smoking at the smoking cessation clinic had nearly 3 times higher hazard rates compared to those seen more than once at the smoking cessation clinic whose records indicated abstinence on at least two of their 3-, 6- or 12-month follow-up visits) [Hazard ratio: 2.7; 95%CI = 1.4-5.0]	Smoking cessation therapy is a cost-effective means of improving of improving smoking abstinence and cessation, cancer treatment, and health outcomes among cancer patients following diagnosis

Between June 2009 and May 2012, a longitudinal study was conducted to evaluate smoking cessation among 201 cancer patients visiting an outpatient smoking cessation clinic in a cancer centre in Jordan. The prevalence of smoking abstinence was 23.4% in three months, and 6% in one year. Participants were unwilling to abstain from smoking because they were either unable to handle withdrawal, saw no value in quitting, and/or were experiencing depression [12].

To investigate the status of quitting and examine the motivations for tobacco cessation after receiving a head and neck cancer diagnosis, Matthew and colleagues conducted a cross-sectional study among 71 cancer patients aged ≥ 25 years who had a history of tobacco smoking before their cancer diagnosis. Overall, oncologists’ advice helped 67.6% of the patients quit smoking after the cancer diagnosis [13].

To evaluate receipt of care at a smoking cessation clinic and the effect of assisted abstinence through the smoking cessation clinic on short-term (two-year) survival after a cancer diagnosis, a retrospective cohort study was conducted among 3,403 cancer patients diagnosed between 2009 and 2016. These patients were cigarette smokers and had received treatment at King Hussein Cancer Center in Jordan. Hawari and colleagues reported a 20% smoking cessation rate among cancer patients seen at the smoking cessation clinic. The study also reported higher hazard rates among cancer patients that had never been seen at the smoking cessation clinic or seen after a year from diagnosis compared to those seen more than once at the smoking cessation clinic whose records indicated abstinence on at least two of their 3-, 6- or 12-month follow-up visits [14].

## Discussion

Findings from this scoping review revealed that smoking cessation advice delivered by physicians helped cancer patients to quit smoking. A cancer diagnosis is sometimes regarded as a teachable moment—a sporadic occasion in a person’s life when significant behavioral change is possible [15]. Regardless of the specialty of the physicians involved i.e., oncologists or general practitioners, our study found that the physician-patient interaction created an opportunity to initiate smoking cessation counseling sessions between the parties. From this scoping review, the prevalence of smoking cessation ranged between 2% and 44% in LMICs. This rate (36%) is not like 2–44 reported in the National Health and Nutrition Examination Survey study, as cited in literature [16, 17]. A cross-sectional study conducted among cancer patients at Mary Babb Randolph Cancer Center, West Virginia University Hospital, United States reported that 62% of smokers received smoking cessation counseling from their doctors, and 44% of these tobacco users quit smoking [18].

From our current review, it was found that nearly 60% of cancer patients were willing to quit smoking after a diagnosis of cancer. However, discussions and prescriptions for medications to quit smoking were not frequently provided by physicians. Unlike HICs, there is a mass emigration of physicians and an increased workload for physicians currently working in LMICs [18]. To handle the demands of the health facility, physicians in LMICs may be likely to omit information on smoking cessation during counseling and oncology care. Strategies such as greater rewards for labor may be required for physician retention in LMICs to increase the potential for implementing physician-brief advice for smoking cessation in oncology care settings.

The finding that cancer patients who smoked fewer cigarettes were more likely to quit smoking compared to heavy smokers carries significant implications for both healthcare providers and public health interventions. Light smokers may find it comparatively easier to quit due to a lower level of nicotine dependence. Similar to previous studies, heavy smokers are at a higher risk of nicotine dependence and it is also difficult for them to quit smoking [15, 16]. Heavy smokers, on the other hand, may feel more in control of their habit and be more successful in their quit attempts. Doctors ought to strongly recommend that patients cease smoking and employ motivational interviewing methods for those who are not yet prepared to quit. When interacting with unmotivated patients, clinical engagements should underscore the benefits and significance of quitting, along with the hazards of smoking and expected obstacles to abstaining [17–19]. Therefore, physician-brief smoking cessation advice should be integrated into routine oncology consultations. Physicians should be encouraged to discuss smoking cessation during every patient’s visit, emphasizing the importance of quitting, especially for cancer patients. This can become a standard part of the medical protocol. Culturally sensitive and locally relevant educational materials and messages about the risks of smoking and the benefits of quitting should be designed for physician’s use.

Our current review identified that smoking cessation interventions could consist of many different methods, including the use of motivational messages, teachings, and stress management discussions. Through these approaches, cancer patients could be made comfortable, and able to absorb the lessons from the physician-patient interaction. Cancer Centre, Ontario has launched several initiatives including the use of lectures, blog postings, the production of videos, posters, and patient handouts [17]. It has been emphasized that interventions should be quick and adhere to a script informing patients that giving up smoking is one of the most crucial things they can do to ensure the best outcomes from their cancer treatment. Physicians are urged to quickly introduce the patient to a suitable smoking cessation resource located at the cancer center, hospital, or local area. It must be noted that, when speaking to their patients directly about the value of quitting smoking, oncologists and other categories of physicians play a crucial role in inspiring them. When they don’t, all other healthcare professionals trying to assist the patient in quitting smoking are put in jeopardy.

Our study suggests that certain conditions may impede the opportunity that exists in using smoking as a teachable moment to counsel lung cancer patients to stop tobacco use. Self-efficacy toward quitting smoking is a core strategy required to fast-track the smoking cessation process among cancer patients [20]. However, when the self-efficacy towards quitting smoking is low, cancer patients lack the belief in their ability to cope with the stress and symptoms of withdrawal and their ability to resist the temptation to resume smoking [21]. Text message-based interventions and mobile apps can enhance patients’ self-efficacy to quit smoking. These can include motivational messages, progress tracking, and even direct communication with healthcare providers through telemedicine services.

Barriers to the effective implementation of physician-brief advice for smoking cessation include limited time and resources, minimal patient-physician contact, and physicians’ perception that patients are too stressed to engage in smoking cessation discussions [13]. Other studies have reported low levels of education, unemployment, easy access to tobacco in a hospital setting, and lack of knowledge about smoking cessation strategies as barriers to effective smoking cessation [22, 23]. Positive and supportive communication, devoid of judgment, is required for cancer patients to deal with stress during physician-brief advice delivery. Peer support can be a powerful motivator to address some of the barriers. It is important to establish peer support groups where current or former cancer patients who have successfully quit smoking can share their experiences and provide encouragement to those trying to quit.

### Limitations

This study was limited to data from cancer patients living in LMICs and as such, reflects the perceptions and experiences in health facilities where smoking cessation counseling is organized for patients receiving oncology care only. The authors acknowledge that grey literature was not included in the review, and that this limitation could have hindered the comprehensiveness of findings. Future research may be undertaken to obtain and compare the pooled prevalence of smoking cessation due to physician-brief advice delivered to cancer patients in both LMICs and HICs. The sociodemographic characteristics of the participants in the primary studies were not reported, and this could have masked some core influencers of smoking cessation among cancer patients. The use of few data repositories (based on the exploratory analysis conducted before the review) could have limited the yield from literature search.

### Strengths

The methodological strengths of this scoping review are the systematic search process employed, and the inclusion of studies conducted over a substantial time frame. Thus, these strengths contribute to the reliability of findings. Heterogeneity allowed the inclusion of studies with diverse methodologies, interventions, and research questions. This inclusivity provided a more comprehensive understanding of the topic, enabling a wider exploration of perspectives and approaches.

## Conclusion

Physician-brief advice is an important intervention to promote smoking cessation among cancer patients. However, this intervention is rarely implemented for cancer patients in LMICs. To bridge this gap in clinical practice, physicians must receive training to deliver brief advice for smoking cessation. Potential strategies for physicians’ training regarding smoking cessation interventions include online training modules and webinars, and integration of smoking cessation education into the medical curriculum. Policymakers should allocate the resources necessary to implement Physician-brief advice, and design training programs for physicians focusing on physician-brief advice tailored to cancer patients. The National Association of Medical Practitioners and the Ministry of Health in each LMIC should integrate physician-brief advice into cancer care to ensure that this becomes the standard practice in oncology settings.

## Data Availability

All data generated or analyzed during this study are available upon reasonable request from the corresponding author.

## Abbreviations

HIC: High-Income Countries
LMICs: Low Middle-Income Countries
RCTs: Randomized Controlled Trials
SC: Smoking Cessation
